# Immunotherapeutic Targeting of Mesothelin Positive Pediatric AML Using Bispecific T Cell Engaging Antibodies

**DOI:** 10.3390/cancers13235964

**Published:** 2021-11-26

**Authors:** Anilkumar Gopalakrishnapillai, Colin E. Correnti, Kristina Pilat, Ida Lin, Man Kid Chan, Ashok D. Bandaranayake, Christopher Mehlin, Anne Kisielewski, Darcy Hamill, Allison J. Kaeding, Soheil Meshinchi, James M. Olson, Edward Anders Kolb, Sonali P. Barwe

**Affiliations:** 1 Nemours Centers for Childhood Cancer Research & Cancer and Blood Disorders, Alfred I. duPont Hospital for Children, Wilmington, DE 19803, USA; anil.g@nemours.org (A.G.); AKisielewski@hygiena.com (A.K.); Darcy.Hamill@nemours.org (D.H.); eakolb@nemours.org (E.A.K.); 2 Clinical Research Division, Fred Hutchinson Cancer Research Center, Seattle, WA 98109, USA; ccorrent@fredhutch.org (C.E.C.); kpilat@fredhutch.org (K.P.); ilin2@fredhutch.org (I.L.); mchan2@fredhutch.org (M.K.C.); abandara@fredhutch.org (A.D.B.); cmehlin@fredhutch.org (C.M.); akeading@fredhutch.org (A.J.K.); smeshinc@fhcrc.org (S.M.); jolson@fredhutch.org (J.M.O.)

**Keywords:** bispecific T cell engaging antibodies, mesothelin, pediatric acute myeloid leukemia, patient-derived xenograft models, immunotherapy

## Abstract

**Simple Summary:**

Immunotherapy development in pediatric AML has been slow due to the paucity of validated AML-specific targets. We recently identified mesothelin (MSLN) as a therapeutic target in pediatric AML. Mice receiving T cell engaging bispecific antibodies (BsAbs) targeting MSLN and CD3 achieved complete remission and durable responses in two MSLN-positive patient-derived xenograft (PDX) models. This is a first report showing MSLN-targeting BsAbs are a viable immunotherapy for MSLN-positive pediatric AML.

**Abstract:**

Advances in the treatment of pediatric AML have been modest over the past four decades. Despite maximally intensive therapy, approximately 40% of patients will relapse. Novel targeted therapies are needed to improve outcomes. We identified mesothelin (MSLN), a well-validated target overexpressed in some adult malignancies, to be highly expressed on the leukemic cell surface in a subset of pediatric AML patients. The lack of expression on normal bone marrow cells makes MSLN a viable target for immunotherapies such as T-cell engaging bispecific antibodies (BsAbs) that combine two distinct antibody-variable regions into a single molecule targeting a cancer-specific antigen and the T-cell co-receptor CD3. Using antibody single-chain variable region (scFv) sequences derived from amatuximab-recognizing MSLN, and from either blinatumomab or AMG330 targeting CD3, we engineered and expressed two MSLN/CD3-targeting BsAbs: MSLN^AMA^-CD3^L2K^ and MSLN^AMA^-CD3^AMG^, respectively. Both BsAbs promoted T-cell activation and reduced leukemic burden in MV4;11:MSLN xenografted mice, but not in those transplanted with MSLN-negative parental MV4;11 cells. MSLN^AMA^-CD3^AMG^ induced complete remission in NTPL-146 and DF-5 patient-derived xenograft models. These data validate the in vivo efficacy and specificity of MSLN-targeting BsAbs. Because prior MSLN-directed therapies appeared safe in humans, MSLN-targeting BsAbs could be ideal immunotherapies for MSLN-positive pediatric AML patients.

## 1. Introduction

Survival in children with acute myeloid leukemia (AML) remains poor, with very few advances in therapy over the past 40 years. Despite maximally intensive therapy, approximately 40% of children will relapse. Nearly a third of newly diagnosed children and all children following relapse require myeloablative therapy with hematopoietic stem cell transplantation. Efforts to improve survival and reduce toxicities must focus on novel targeted immunotherapies that can ultimately replace the highly toxic standard of care.

In the past three years, multiple therapies were approved for adults with AML. The AML that occurs in children, however, is different from adult AML. It is unlikely that targeted therapies in adults will translate to effective therapies in children because the genetic and epigenetic events that drive leukemogenesis in children are distinct [1,2,3]. Therefore, it is necessary to develop therapies specific for pediatric disease. The recent successful development of immunotherapies for children with acute lymphocytic leukemia (ALL) is promising [4], however immunotherapeutic development in AML has been slow due to the paucity of validated AML-specific targets, which are critical for a successful targeting strategy [5]. Immunotherapeutic targeting of CD123 and CD33 is encouraging, but the occurrence of on-target, off-tumor effects in normal tissues, especially hematopoietic stem cells, is of concern [6]. 

Mesothelin (MSLN) was first identified as one of the seven genes overexpressed in pediatric AML patients compared with healthy bone marrow specimens [7]. We and others have further verified that MSLN is highly expressed on the cell surface of a subset of pediatric AML samples predominantly belonging to patients with KMT2A-rearrangements and core binding factor AML [8,9]. While MSLN expression was observed in nearly one third of pediatric patients, only 11% of adult AML patients showed MSLN expression 8. MSLN is a 40 kDa glycosyl phosphatidyl inositol-anchored membrane protein that is overexpressed in a variety of epithelial tumors [10]. It has been utilized as an immunotherapy target in solid tumors in the form of a chimeric, high affinity monoclonal antibody (amatuximab) [11], an antibody drug conjugate (anetumab ravtansine) [12], an antibody Thorium227 conjugate (MSLN-TTC) [13], an immunotoxin (SS1P) [14], chimeric antigen receptor-T cells [15] and T cell engaging bispecific antibodies in IgG-based formats [16]. It is anticipated that immunotherapeutic targeting of MSLN in pediatric AML will be safe and highly beneficial because (1) MSLN function in normal tissues is dispensable [10], (2) MSLN is expressed only on the luminal surface of non-neoplastic cells in the body, a space that is minimally exposed to antibodies in the bloodstream, and (3) MSLN is not expressed in normal bone marrow tissue in children [7,17]. 

Broadly speaking, T cell-engaging bispecific antibodies (BsAbs) combine the variable regions of multiple antibodies, often as single chain variable fragment (scFv) domains targeting a cancer-specific antigen and (typically) the epsilon subunit of the T cell co-receptor CD3 to induce tumor cell lysis by effector T cells. The early success of blinatumomab, a bispecific T cell engager targeting CD19 and CD3 for relapsed/refractory ALL [18], suggests that BsAbs directed against myeloid-specific antigens could similarly transform clinical outcomes in pediatric AML. In this study, we designed a series of proof-of-concept BsAbs using published sequences to determine if a T cell redirecting therapy could effectively target MSLN-positive AML. We tested the specificity and efficacy of the MSLN-targeting BsAbs in vitro and in disseminated xenograft models of KMT2A-rearranged pediatric AML, because it is one of the major subtypes with predominant MSLN expression [8]. The MSLN/CD3-targeting BsAbs were effective in promoting T cell activation and eliminating leukemic burden in mice engrafted with MSLN expressing AML. The T cell expansion and anti-leukemic effect was not observed in mice receiving a non-specific BsAb or those engrafted with MSLN-negative AML. Our data validate the in vivo efficacy of MSLN-targeting BsAbs and highlight MSLN as a novel target for immunotherapy in pediatric AML.

## 2. Materials and Methods

### 2.1. Cell and Patient-Derived Xenograft Lines

MV4;11 and OVCAR-3 cells were obtained from American Type Culture Collection (Manassas, VA, USA). C30 (A2780) cells were obtained from Sigma (St. Louis, MO, USA). MV4;11 cells were cultured in IMDM supplemented with 10% fetal bovine serum as described previously [19]. OVCAR-3 and C30 cells were cultured in RPMI-1640 supplemented with 10% fetal bovine serum. FreeStyle™ 293-F cells (R79007) were obtained from ThermoFisher Scientific (Waltham, MA, USA). The patient-derived xenograft (PDX) line NTPL-146 was generated by engrafting a *KMT2A-MLLT1* bearing a primary pediatric AML sample collected under an Institutional Review Board approved protocol [20]. The PDX line CBAM-44728-V1 bearing *KMT2A-MLLT10* fusion (referred to as DF-5) was obtained from the Dana Farber Cancer Institute PRoXe depository [21]. Cell lines were authenticated using the AmpFLSTR Identifiler PCR Amplification Kit (ThermoFisher Scientific) and confirmed to be free of mycoplasma contamination (PCR analysis).

### 2.2. Generation of an MV4;11 Cell Line Expressing MSLN (MV4;11:MSLN)

Using lentivirus production methods described previously [22], we generated a lentivirus encoding the full-length human MSLN transgene (residues 1–630) and a cis-acting iRFP reporter downstream of an internal ribosome entry site. Briefly, a 24-well plate was seeded with 0.5 million cells per well at a density of 1 million/mL. Cells were transduced with 250 or 500 μL of 1× lentivirus in the presence of polybrene (4 μg/mL per well) at a final volume of 1 mL. Once cells and virus were plated, the plate was centrifuged at 800× *g* for 1 h. Following centrifugation, the media was aspirated, and 1.5 mL of fresh media was added to each well. Three days later, the cells were checked by flow using a NovoCyte 3000 Flow Cytometer (ACEA Biosciences; San Diego, CA, USA). All cultures that were greater than 85% iRFP positive were combined. Cells were then flow sorted using a BD FACS Aria II (BD Biosciences; Franklin Lakes, NJ, USA) and subsequently expanded. 

### 2.3. Flow Cytometry and Quantitation of MSLN Cell Surface Expression

Anti-human mesothelin antibody (Clone MB, Catalog No. 530203, BioLegend; San Diego, CA, USA) was conjugated with PE/R-phycoerythrin conjugation kit (Abcam, Cambridge, MA, USA; Catalog No. ab102918) per the manufacturer’s instructions. Samples stained with the PE-conjugated anti-MSLN antibody were analyzed on a NovoCyte 3000 Flow Cytometer. MSLN cell surface expression was quantitated using a BD Quantibrite PE Phycoreythrin Fluorescence Quantitation kit (BD Biosciences) following the manufacturer’s protocol.

### 2.4. BsAb Design, Protein Expression and Purification from Human Cells

The amino acid sequences of single-chain variable fragments derived from the MSLN-targeting antibody amatuximab and the CD3-engaging domains of BsAbs targeting CD19 (blinatumomab) or CD33 (AMG 330) were combined into scFv-scFv or IgG-scFv constructs (see Supplementary Table S1). The resulting designs were then reverse translated using human codons, further codon-optimized, and synthesized de novo at GenScript. We utilized the Daedalus mammalian expression platform for the production and purification of secreted BsAb proteins, using methods described previously [22]. The expression system utilizes suspension-adapted FreeStyle 293-F cells and a highly optimized lentiviral transduction protocol to generate cell lines that secrete proteins at high levels. The lentiviral vector contains a cis-linked fluorescent protein reporter driven by an internal ribosome entry site (IRES) that allows for the tracking of relative protein expression levels. All mammalian proteins described in Supplementary Table S1 were purified directly from conditioned media using HisTrap FF Crude columns (#17528601) and subsequently loaded onto a HiLoad 16/600 Superdex 200 pg size-exclusion column (#28989335). Fractions corresponding to the monomeric protein were pooled, flash frozen and further analyzed post-thaw on a Superdex 200 Increase 10/300GL (#28990944) size-exclusion column using an AKTA pure 25 instrument.

### 2.5. T Cell Cytotoxicity 

Healthy, unstimulated human donor peripheral blood mononuclear cells (PBMCs) were obtained from Bloodworks NW (Seattle, WA, USA), frozen and stored in liquid nitrogen. T cells were isolated from a thawed PBMC aliquot using a CD3+ magnetic negative selection kit (Stemcell Technologies, Vancouver, BC, Canada) according to the manufacturer’s instructions and evaluated by flow cytometry to quantify the expression of the markers CD3, CD28, CD4 and CD8 (BioLegend). All further assay preparation and sample collection for readout was automated using a Hamilton Microlab Starlet liquid handler. In a 96-well plate, CD3-engaging antibodies were added at 5-fold dilutions starting at 1000 ng/mL. The MV4;11:MSLN-luc-iRFP target cells were co-cultured with purified T cells at a 5:1 E:T ratio. All conditions were performed in triplicate. Cells were incubated at 37 °C for 48 h. At 24 h, 50 μL of supernatant was collected to screen for cytokine release. At 48 h, 35 μL of cells were transferred to a new 96-well, v-bottom plate and mixed with DAPI to 1× final concentration. Samples were run on an iQue Screener Plus (Intellicyt, Sartorius, Wood Dale, IL, USA) and live target cells were identified by forward/side scatter distribution, negativity for DAPI, and positive iRFP expression using accompanying ForeCyt software. Raw cell counts were exported to Excel (Microsoft) and percent cytotoxicity was calculated utilizing the following formula:

(1) $$\%\;Cytotoxicity=\frac{Mean\;count\;of\;live\;{iRFP}^{+}\;cells\;\left(no\;drug\right)-count\;of\;live\;{iRFP}^{+}\;cells\;\left(w/\;drug\right)}{Mean\;count\;of\;live\;{iRFP}^{+}\;cells\;\left(no\;drug\right)}$$

Fitted curves were generated by applying a Sigmoidal, 4PL, x is log(concentration) model using GraphPad Prism 7.

### 2.6. Cytokine Detection and Quantification

IL-2, IFNγ, and TNFα were quantified using the QBeads PlexScreen human 3-Plex reagent kit (Sartorius) with modifications to the manufacturer’s instructions. PlexScreen cytokine standards were resuspended in Assay Buffer to a concentration of 5000 pg/mL and diluted twofold in CTL media (RPM1640, 10% human serum, 4 mM Glutamax, 100 U/mL penicillin/streptomycin, 50 μM β-mercaptoethanol) to generate the top concentration of a 12-point standard curve. The standard curve dilution and all reagent addition steps were completed on a Hamilton Starlet liquid handler. In a 96-well format, 10 μL of T cell killing assay supernatant collected at 24 h or standard was incubated with 10 μL of the prepared capture beads cocktail. The samples were shaken at 800 rpm for 1 h at room temperature. 10 μL of detection cocktail was added and incubated with shaking for 2 h at room temperature. The samples were washed with the Wash Buffer reagent and resuspended to a final volume of 30 μL of Wash Buffer. The cytokines were analyzed on the iQue Screen Plus flow cytometer using the PE fluorescence channel.

### 2.7. Leukemia Xenograft Models

Female 4–6-week-old NSG-SGM3 mice were intravenously injected with 5 × 10^6^ MV4;11, 5 × 10^6^ MV4;11:MSLN, or 2 × 10^6^ DF-5 cells. For the NTPL-146 study, 3 × 10^6^ cells were injected in female, 4–6-week-old NSG-B2m mice via the tail vein. At indicated time points, mice were randomly assigned to treatment groups. Leukemia progression was assessed by evaluating the percentage of human chimerism in peripheral blood by flow cytometry as described previously [20]. Human peripheral blood pan-T cells (StemCell Technologies; Cambridge, MA, USA) were quickly thawed, resuspended in phosphate buffered saline, and injected via the tail vein (3 × 10^6^ cells per mouse). Purified BsAb proteins were administered via intraperitoneal injections at 3 mg/kg daily. Chemotherapy consisted of 3 doses of 1.5 mg/kg daunorubicin i.v. and 5 doses of 50 mg/kg cytarabine i.p. Mice were monitored daily and euthanized when any of the experimental endpoints such as reduced mobility, weight loss greater than 20% of body weight, hind limb paralysis, or distended abdomen were met. Postmortem analysis was conducted to determine the terminal bone marrow leukemic load. Femurs were harvested and flushed bone marrows were stained with mouse CD45 (BioLegend #103112), human CD45 (BioLegend #304029) and human CD3 (BioLegend #317306) following incubation with Human BD Fc Block. Samples were evaluated by flow cytometry for the percentage of AML cells (human CD45+CD3−) and T cells (human CD45+CD3+). The percentage of AML cells or T cells was calculated using the following formula.

(2) $$\%\;Human\;cells=\frac{Human\;cell\;count}{Human\;cell\;count+\;Mouse\;cell\;count}\times 100$$

Investigators were not blinded to the treatment groups. All procedures involving mice were approved by the Nemours Institutional Animal Care and Use Committee (approval number - RSP19-09761-001). 

### 2.8. Statistical Analysis

GraphPad Prism 7 was used for statistical analysis. *p* values were calculated using two-tailed Student’s T-test (two-samples, with unequal variance) for determining the statistical significance of the difference between two groups. For Kaplan-Meier survival curves, *p* values were calculated using the Log-rank (Mantel–Cox) test. Pilot in vivo experiments were conducted to determine the appropriate sample size needed to provide 80% power at 95% confidence using statistical power analysis to detect significant differences in qualitative (survival) and quantitative (% AML cells) parameters in compliance with ARRIVE guidelines [23].

## 3. Results

### 3.1. Design and Expression of BsAbs Co-Targeting MSLN and CD3

T cell engaging BsAbs targeting MSLN and CD3 were engineered by combining scFv sequences directed against MSLN derived from amatuximab (also known as MORAb-009) [24], and those targeting CD3 derived from two well-known T cell engaging BsAbs—blinatumomab (MSLN^AMA^-CD3^L2K^) and AMG 330 (MSLN^AMA^-CD3^AMG^) (Supplementary Table S1). The affinities of these targeting antibodies for human MSLN and CD3 were determined previously: Amatuximab (KD~1.5 nM) [25], L2K (KD~260 nM) [26] and AMG 330 (KD~5.1 nM) [27]. These BsAbs were formatted as tandem scFv-scFv molecules by arranging the variable light (VL) and heavy (VH) domains in the VLVH-VHVL orientation, similar to blinatumomab, and expressed in HEK293 FreeStyle cells (Supplementary Table S1). We also engineered an IgG-scFv by fusing the L2K scFv (in the VHVL orientation) to the carboxy terminus of the light chain of amatuximab to generate a BsAb with an improved half-life. Each protein secreted at high levels and was easily purified using affinity and size exclusion chromatography (Supplementary Figure S1). The final purified products used for all in vitro and in vivo studies are shown in the SEC insets. 

### 3.2. Evaluation of In Vitro Activity of MSLN-Targeting BsAbs 

**Figure 1:**
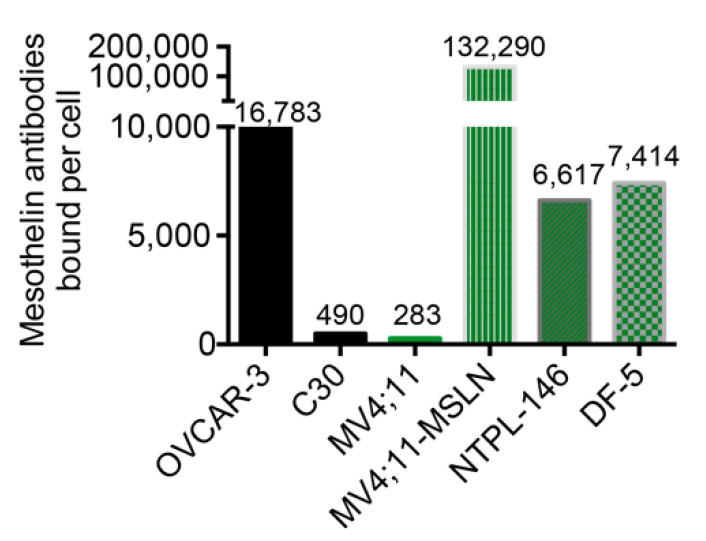
MSLN cell surface expression and quantitation in AML cell and PDX lines. Quantitation of MSLN antibodies bound per cell using Quantibrite. Ovarian carcinoma cell lines OVCAR-3 (MSLN-positive) and C30 (MSLN-negative) were used for reference.

MV4;11 is a pediatric AML cell line that does not constitutively express MSLN. Quantification of MSLN cell surface expression in MV4;11 cells using Quantibrite detected <300 MSLN antibodies bound per cell, comparable to the MSLN-negative ovarian carcinoma cell line C30 (Figure 1). 

**Figure 2:**
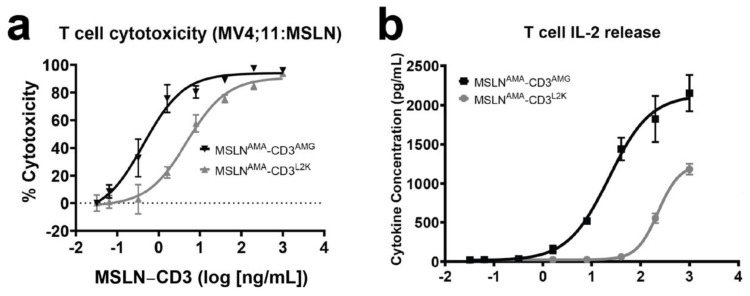

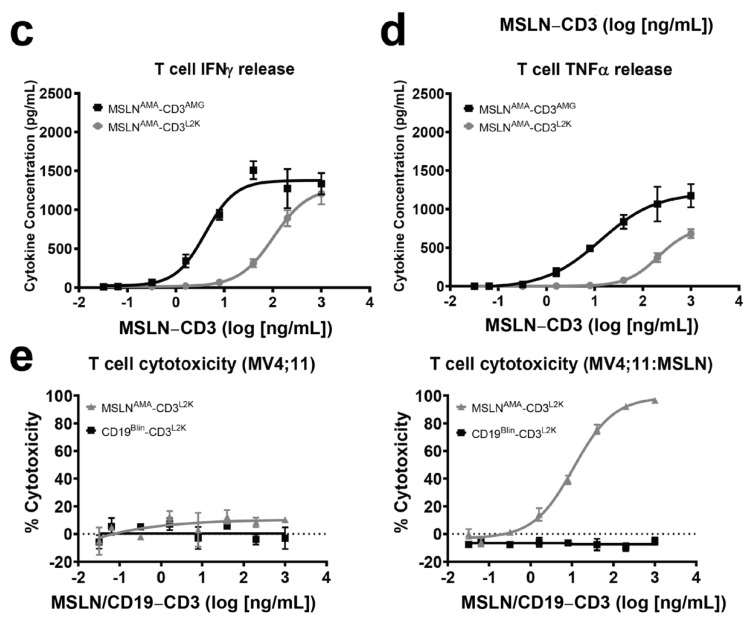
T cell killing and cytokine profiling of two scFv-scFv T cell engagers. (**a**) Representative T cell-mediated cytotoxicity for each of the BsAbs purified in Supplementary Figure S1. Each curve represents one BsAb at multiple concentrations (pg/mL) with three technical replicates. Data are shown as means ± SD. (**b**–**d**) Analysis of T cell cytokine secretion after 24 h for IL-2, IFNγ and TNFα as a function of BsAb concentration. Each curve represents cytokine quantitation at multiple BsAb concentrations (pg/mL) with technical replicates. Data are shown as means ± SD. (**e**) Representative T cell-mediated cytotoxicity for MSLN-specific BsAb and a control BsAb in MV4;11 (left) and MV4;11:MSLN cells (right). Data from three technical replicates are plotted as means ± SD.

The MV4;11:MSLN cell line generated by lentiviral transduction of MV4;11 cells using human MSLN had high MSLN expression with 132,290 anti-MSLN antibodies bound per cell, which was far greater than OVCAR-3, a MSLN-positive ovarian carcinoma cell line (Figure 1). To assess the in vitro potency of the two BsAbs constructed with different CD3-engaging scFvs, MV4;11:MSLN cells were co-cultured with freshly isolated naïve T cells (CD4 and 8) at an effector to target (E:T) ratio of 5:1 and increasing concentrations of BsAbs. Both MSLN-targeting BsAbs induced T cell mediated cytotoxicity of MV4;11:MSLN cells analyzed at 48 h post treatment (Figure 2a). 

The MSLN^AMA^-CD3^AMG^ was more effective in inducing cell death of MV4;11:MSLN cells with EC50 values for MSLN^AMA^-CD3^L2K^ and MSLN^AMA^-CD3^AMG^ being 4.82 ng/mL (91.30 pM) and 0.43 ng/mL (8.12 pM), respectively. Cytotoxicity mediated by MSLN-targeting BsAbs was accompanied by a dose-dependent induction of cytokine production (Figure 2b–d). Cytokine release triggered by MSLN^AMA^-CD3^AMG^ was greater than that induced by MSLN^AMA^-CD3^L2K^, and the EC50 values were lower by more than a log for MSLN^AMA^-CD3^AMG^ versus MSLN^AMA^-CD3^L2K^, likely due to the differences in affinity and epitope between the two CD3 clones. T cell mediated cytotoxicity was also assessed against parental MV4;11 (Figure 2e, left panel) and MV4;11:MSLN (Figure 2e, right panel) cells using both MSLN^AMA^-CD3^L2K^ and CD19^Blin^-CD3^L2K^, as a CD3 matched pair, confirming the specificity of these BsAbs. 

### 3.3. Testing MSLN-Targeting BsAbs in Cell Line-Derived Xenograft Models with or without MSLN Expression 

**Figure 3:**
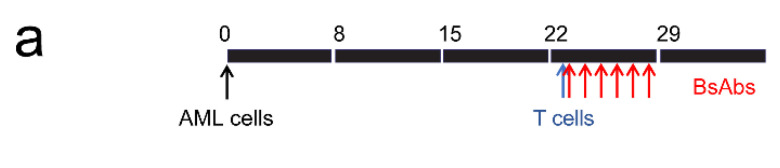

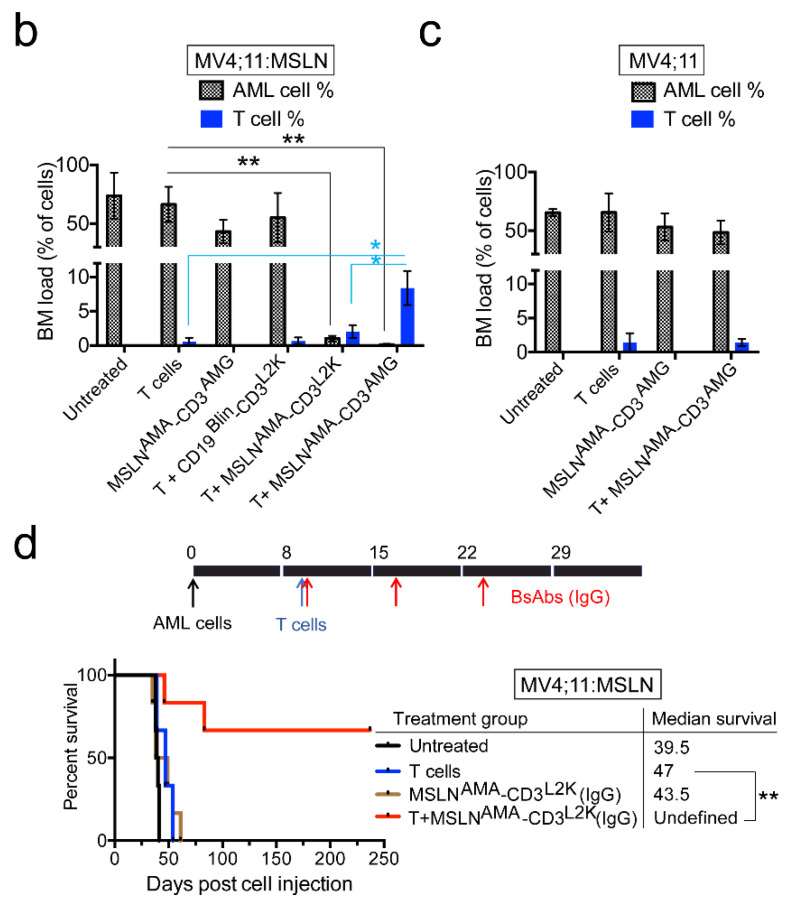
Efficacy of MSLN-targeting BsAbs in MV4;11:MSLN and MV4;11 xenograft models. (**a**) Schematic showing the dosing scheme for BsAbs. (**b**,**c**) Mice were euthanized when they reached pre-determined experimental endpoints. Bone marrow was flushed and stained with mouse specific CD45-APC, human specific CD45-Pacific blue and human specific CD3-FITC antibodies. Graph shows mean AML and T cell percentages in the bone marrow of mice engrafted with MV4:11:MSLN cells (**b**) or MV4;11 cells (**c**). Error bars denote SE of the mean. *n* = 5 per group. * *p* < 0.05, ** *p* < 0.005. (**d**) Schematic showing the dosing scheme for the IgG-based BsAb. Kaplan-Meier survival plot showing the median survival (** *p* < 0.01).

MV4;11 and MV4;11:MSLN isogenic cell lines provide a tool to evaluate the specificity and efficacy of MSLN-targeting BsAbs. Each of these cell lines (5 × 10^6^) was injected intravenously into NSG-SGM3 mice to generate disseminated xenograft models. Peripheral blood was sampled on days 15 and 22 (post implantation) to determine the percentage of chimerism. Once engraftment was confirmed (day 23, >0.4% AML cells in peripheral blood), mice were injected with 3 × 10^6^ naïve allogeneic human T cells to serve as effector cells. Mice (*n* = 5 per arm) were then treated daily for 6 days with MSLN^AMA^-CD3^L2K^ or MSLN^AMA^-CD3^AMG^ at a well-tolerated dose of 3 mg/kg shown to be effective for other BsAbs (Figure 3a). Because MV4;11 cells do not express CD19 [28], a BsAb CD19^Blin^-CD3^L2K^ was used as a non-specific control antibody. A cohort of mice (*n* = 5 per group) that were untreated or received either MSLN^AMA^-CD3^AMG^ treatment alone or T cell infusion only served as controls. 

Mice were euthanized when they developed reduced mobility, weight loss greater than 20% of body weight, hind limb paralysis, or distended abdomen. In this particular model, the mice typically become moribund due to extramedullary chloromas. The bone marrow was analyzed postmortem to assess and compare disease burden in the marrow, which is the primary concern in human pediatric AML patients. In the presence of T cells, MSLN-targeting BsAbs MSLN^AMA^-CD3^AMG^ and MSLN^AMA^-CD3^L2K^ had significantly lower mean ± SE AML cell percentage in the bone marrow (0.1 ± 0.0% and 1.0 ± 0.2% respectively) compared to T cells alone (66.4 ± 6.7%) (Figure 3b; ** *p* < 0.005). Consistent with the lack of CD19 expression in MV4;11:MSLN cells (data not shown), the mean AML cell load in the bone marrow of mice treated with non-specific BsAb CD19Blin-CD3L2K (55.0 ± 9.4%) was not significantly different from that of T cells alone (66.4 ± 6.7%). These results showed near-complete eradication of marrow disease even as mice developed life threatening, model-specific, extramedullary disease. 

MSLN^AMA^-CD3^AMG^ was more effective than MSLN^AMA^-CD3^L2K^ (*p* < 0.05), mean bone marrow load in mice treated with MSLN^AMA^-CD3^L2K^ (1.0 ± 0.2%) was ten-fold higher than that in MSLN^AMA^-CD3^AMG^ (0.1 ± 0.0%) (Figure 3b), consistent with our in vitro results (Figure 2a). The differences in the mean AML load in the bone marrow of mice treated with BsAb MSLN^AMA^-CD3^AMG^ alone (43.3 ± 4.4%), untreated (73.7 ± 8.8%) or T cell alone (66.4 ± 6.7%) groups were not statistically significant, confirming that BsAb molecules or human T cells alone were ineffective. Mice treated with MSLN^AMA^-CD3^AMG^ survived significantly longer than any other treatment group, suggesting that extramedullary disease was also reduced by these agents, though extramedullary disease burden is much more difficult to quantify than that in marrow. These mice showed an 11-day improvement in median survival compared to untreated mice and a 9-day extension of median survival compared to the T cells alone group (* *p* < 0.05, Supplementary Figure S2a). 

Bone marrow from mice treated with MSLN^AMA^-CD3^AMG^ and T cells had 8.4 ± 1.4% human T cells, which was significantly greater than mice receiving MSLN^AMA^-CD3^L2K^ with T cells (2.1 ± 0.4%), T cells only (0.6 ± 0.2%), or CD19Blin-CD3L2K with T cells (0.7 ± 0.2%) (Figure 3b; * *p* < 0.05). Coupled with superior cytokine release data in vitro, these data support the conclusion that MSLN^AMA^-CD3^AMG^ causes superior T cell expansion, likely due to cytokine release, compared to MSLN^AMA^-CD3^L2K^ and that this leads to lower marrow disease burden and improved survival. 

The more effective BsAb MSLN^AMA^-CD3^AMG^ (with or without T cells) was then tested in mice transplanted with parental MV4;11 cells that lack MSLN expression (n = 5 per group). The mean AML bone marrow load of mice receiving MSLN^AMA^-CD3^AMG^ treatment along with T cell infusion (48.6 ± 5.7%) was not significantly different than mice receiving BsAb alone (53.2 ± 6.7%) or T cells alone (65.6 ± 9.4%) (Figure 3c). The mean T cell percentage in these mice was also comparable to mice receiving T cells alone (1.5 ± 0.3 and 1.4 ± 0.5% respectively). Taken together, these data highlight the specificity of the MSLN^AMA^-CD3^AMG^ BsAb and confirm the mechanism of action. 

We further evaluated the cause for non-clearance of extramedullary chloromas by BsAb treatment. Immunohistochemical analysis of extramedullary chloromas showed high MSLN expression and T cell infiltration (Supplementary Figure S2b), confirming the presence of antigen and effector cells. Because extramedullary chloromas were palpable about 5–7 days before the mice succumbed to disease, the BsAb treatment window (days 23–28) was likely not sufficient to clear the extramedullary disease, owing to the short half-life of BsAbs. To address this difficulty, we produced a MSLN^AMA^-CD3^L2K^ IgG-based BsAb with a prolonged in vivo half-life. Four of the six mice treated with three doses of IgG-based BsAb were disease-free (Figure 3d; ** *p* < 0.01) with bone marrow load less than 0.05% at the time of euthanasia on day 250, indicating that IgG-based BsAb treatment prevented extramedullary chloroma formation in the majority of mice. 

### 3.4. Testing MSLN-Targeting BsAbs in Patient-Derived Xenograft Models 

**Figure 4:**
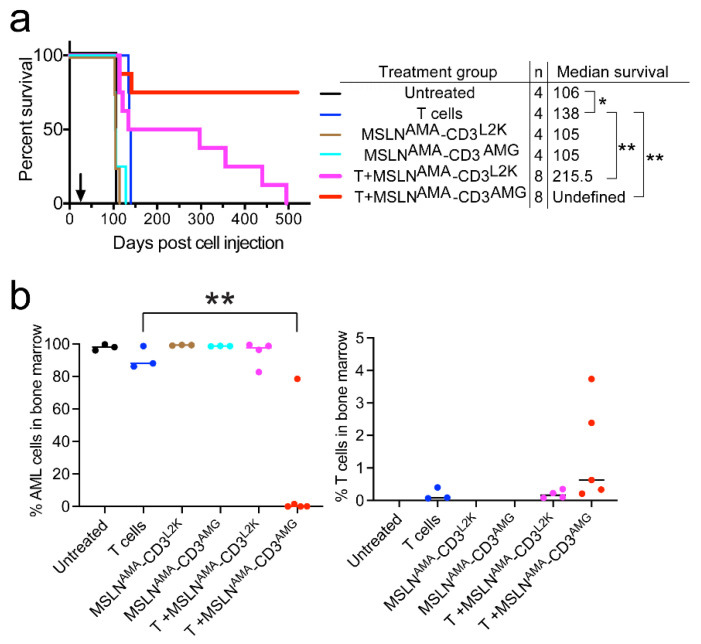
Validation of BsAb efficacy in NTPL-146, a MSLN-positive PDX model. (**a**), Kaplan–Meier survival plot showing the median survival. * *p* < 0.05; ** *p* < 0.001. Arrow indicates time when treatment began. (**b**), Terminal bone marrow AML load (CD45+CD3−) and T cell counts (CD45+CD3+) were plotted. Each dot represents data from a single mouse. Horizontal line indicates the median value. Note that mice were euthanized when they met the experimental endpoint or at the end of the experiment in case of surviving mice belonging to the T + MSLN^AMA^-CD3^AMG^ group, ** *p* < 0.01.

MV4;11:MSLN is a highly aggressive cell line with artificially high MSLN expression and a tendency to develop extramedullary chloromas. The efficacy of MSLN^AMA^-CD3^L2K^ and MSLN^AMA^-CD3^AMG^ was further evaluated in two patient-derived xenograft (PDX) models of pediatric AML with endogenous MSLN expression. NTPL-146 showed uniform MSLN expression that was quantitated at 6617 MSLN antibodies bound per cell (Figure 1). Mice injected with 3 × 106 NTPL-146 cells were randomly assigned to treatment groups (n = 4–8 per group) when human cells were detectable in mouse blood (day 26). A Kaplan–Meier survival plot based on the time when each mouse reached the experimental endpoint showed that 6/8 mice receiving MSLN^AMA^-CD3^AMG^ and T cells survived disease-free until the end of the experiment at day 520, whereas all the mice in the control groups had died by day 138 (Figure 4a). The AML bone marrow load of MSLN^AMA^-CD3^AMG^-treated mice was less than 0.01% at 520 days, whereas the bone marrow load of mice from the other treatment groups was greater than 90% at the time of death, consistent with marrow failure as the proximal cause of death (Figure 4b, Supplementary Figure S3). These data show that the treatment with MSLN^AMA^-CD3^AMG^ is curative in the vast majority of subjects.

Treatment with MSLN^AMA^-CD3^L2K^ accompanied by T cells increased the median survival by 109.5 days compared to untreated mice, while treatment with MSLN^AMA^-CD3^AMG^ showed complete remission in 6/8 mice (** *p* < 0.001 compared to untreated). Thus, the survival benefit of both MSLN^AMA^-CD3^L2K^ and MSLN^AMA^-CD3^AMG^ treated mice greatly exceeded the allogeneic effect T cells alone, which only showed a 22-day improvement in median survival (* *p* < 0.05, compared to untreated). Either MSLN^AMA^-CD3^L2K^ or MSLN^AMA^-CD3^AMG^ treatment alone showed no improvement in survival compared to untreated mice. 

**Figure 5:**
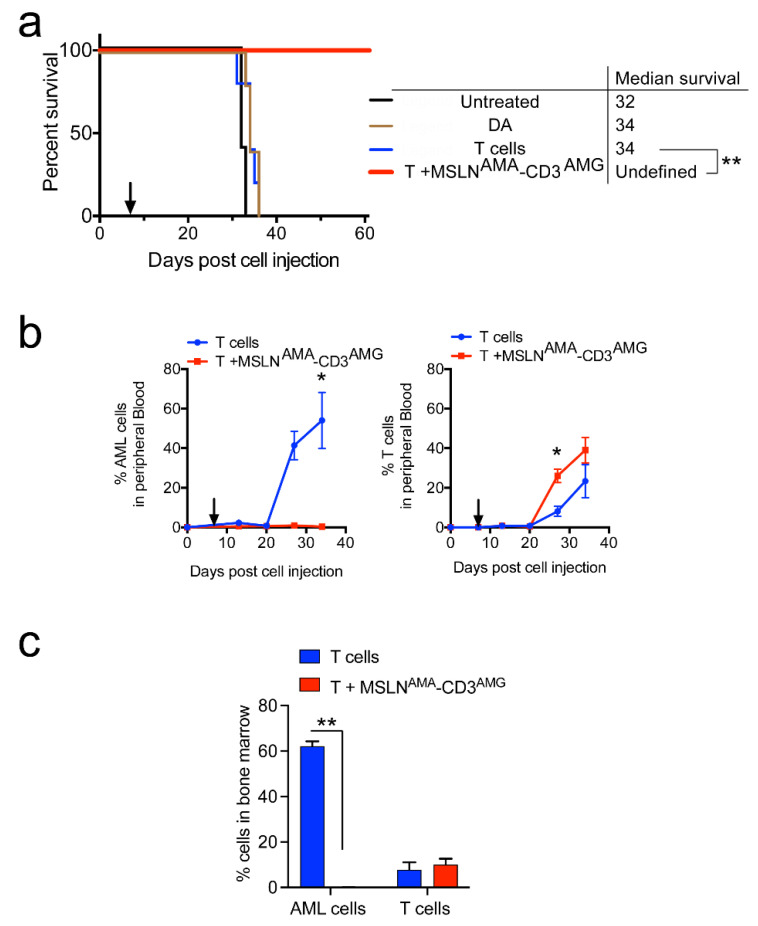
Confirmation of BsAb efficacy in DF-5, a refractory PDX model with MSLN positivity. (**a**) Kaplan–Meier survival plot showing the median survival. *n* = 5 per group, ** *p* < 0.005. (**b**) Graphs show the percentages of AML and T cells in peripheral blood over time. Error bars denote SE of the Mean. * *p* < 0.05. In (**a**,**b**), arrows indicate time when treatment commenced. (**c**) The terminal bone marrow AML (CD45+CD3−) and T cell (CD45+CD3+) percentages upon euthanasia on days 31–36 in mice receiving T cells (when they met experimental endpoint) and in day 61 mice treated with T + MSLN^AMA^-CD3^AMG^ (experiment termination), ** *p* < 0.005.

A second PDX model with endogenous MSLN expression quantitated at 7414 MSLN antibodies bound per cell (Figure 1) was used to test the efficacy of the potent BsAb MSLN^AMA^-CD3^AMG^ in comparison with chemotherapy consisting of daunorubicin and cytarabine (DA). DA treatment, like T cell infusion, did not significantly change median survival compared to untreated mice, while BsAb MSLN^AMA^-CD3^AMG^ in the presence of human T cells was curative (Figure 5a, ** *p* < 0.005). Mice treated with only T cells showed a rapid rise in AML cell percentage in peripheral blood until day 34, when they succumbed to disease, whereas the mice receiving T cells with BsAb had AML cell burdens of less than 1% (Figure 5b; left). Mice treated with BsAbs showed greater expansion of allogeneic human T cells compared to mice receiving T cells alone (Figure 5b, right). Eight weeks post treatment initiation, the surviving mice were euthanized and the AML burden in the bone marrow was evaluated. In contrast with mice receiving T cells alone, mice treated with T cells and BsAbs had no detectable AML cells (Figure 5c, ** *p* < 0.005). Taken together, these data validate the efficacy of MSLN-targeting BsAbs in PDX models with endogenous MSLN expression. 

## 4. Discussion

Development of immunotherapies for pediatric AML patients is an urgent, unmet need. One of the basic requirements to begin to fulfill this need is the identification of suitable targets that are specific to AML blasts in children. Transcriptome analyses identified MSLN as a highly tumor-specific target in greater than 30% of pediatric AML patients [7,8]. Multi-dimensional flow cytometry confirmed cell surface expression [8,9]. We designed two MSLN-targeting BsAbs by fusing the amatuximab scFv domain with the CD3 targeting scFvs from blinatumomab or AMG-330 to determine if BsAbs can effectively target MSLN-positive AML in disseminated xenograft models. Extensive characterization and in vivo evaluation revealed that BsAbs targeting MSLN function in a T cell-dependent and target-specific manner to reduce leukemic burden and prolong survival. 

The immunotherapeutic mechanism underlying improved survival and reduced marrow AML burden was clearly due to joint BsAb and T cell-mediated cancer cell death, since BsAbs alone and T cells alone were ineffective or minimally effective due to an allogeneic effect, respectively. T cells were detectable in bone marrow at the time of euthanasia (up to 520 days, Figure 4b), suggesting that a single dose of T cells is sufficient and long lasting when supported by physiologically relevant cytokine release. 

Because pediatric AML patients with KMT2A rearrangements constitute a subclass with particularly poor prognosis [29,30], our results showing the efficacy of BsAbs in NTPL-146 and DF-5 PDX models with KMT2A rearrangements underscore the significance of BsAbs as immunotherapeutic agents. Moreover, MSLN expression in pediatric AML is predominant in patients with KMT2A-rearranged AML and core binding factor AML, as opposed to other cytogenetic subtypes [8]. Studies are in progress to generate MSLN-positive core binding factor AML PDX models to evaluate the efficacy of BsAbs. 

Almost all antibodies targeting MSLN that are used in clinical or preclinical settings, including in this study, bind the membrane distal region of MSLN, which is shed from the cell surface. This results in a quenching of the therapeutic antibody by soluble MSLN. Previous work showed that the anti-MSLN immunotoxin, SS1(dsFv)PE38, was not effective in killing AML cells in vitro [9]. The authors speculated that this was due to insufficient binding to the cells or because of quenching by soluble MSLN. Similar to blinatumomab, tandem scFv BsAbs used in this study have a short in vivo half-life, necessitating frequent dosing. Our preliminary work with a BsAb in the IgG-based format showed improved survival, highlighting the potential therapeutic benefit of BsAbs with longer in vivo half-life. Experiments are in progress in our laboratories to characterize novel BsAbs with improved half-lives that target epitopes located on the membrane proximal region of MSLN that are preserved on the cell surface. 

The improvement in mouse survival was more pronounced in the NTPL-146 and DF-5 PDX models compared to MV4;11:MSLN engrafted mice. This was likely because the development of extramedullary myeloid sarcomas in the MV4;11:MSLN engrafted mice necessitated euthanasia at early time points, despite the AML burden in the bone marrow being low. Such myeloid sarcomas have been reported in MA9Ras xenografts expressing *KMT2A-MLLT3* fusion protein, limiting the survival efficacy of the IL1RAP antibody, which otherwise showed strong anti-leukemic effect in the bone marrow [31]. Therefore, in the MV4;11:MSLN cell line derived xenograft model, the terminal bone marrow load is a more clinically-relevant measure of efficacy than survival. Nevertheless, MV4;11:MSLN xenografted mice showed improved survival when treated with an IgG-based BsAb, indicating that BsAb treatment not only cleared bone marrow but also prevented extramedullary disease in this model. Likewise, BsAb treatment will likely prevent AML from advancing to extramedullary chloroma formation in pediatric patients, knowing that the likelihood of survival may diminish with the occurrence of extramedullary disease in pediatric patients with certain cytogenetic subtypes [32]. 

MSLN^AMA^-CD3^AMG^ was more efficient in inducing T cell mediated cytotoxicity and cytokine production in vitro compared to MSLN^AMA^-CD3^L2K^. It was also more effective in triggering T cell expansion in the MV4;11:MSLN model, and reducing leukemic burden in vivo in MV4;11:MSLN and NTPL-146 models. This effect was likely due to a combination of factors including the higher apparent affinity of the AMG 330 scFv for CD3ε and differences in epitope recognition between the two CD3 clones. MSLN^AMA^-CD3^L2K^ was designed based on blinatumomab, using a variant of the CD3 clone OKT3 (also known as L2K), which has a lower affinity for CD3 [33] and binds to an epitope on CD3ε that is not conserved in nonhuman primates, while MSLN^AMA^-CD3^AMG^ was designed based on AMG 330 using a proprietary CD3 clone that has a much higher affinity for CD3 [27] and binds a unique epitope on CD3ε that is conserved in nonhuman primates. Different CD3 epitopes within the epsilon subunit are known to possess distinct T cell activation properties depending on affinity and spatial orientation [34,35]. Further studies comparing the in vivo efficacy of these two BsAbs in multiple PDX models are required to confirm the superior response of MSLN^AMA^-CD3^AMG^ in comparison with MSLN^AMA^-CD3^L2K^.

## 5. Conclusions

BsAbs targeting CD123 and CD33 are currently being evaluated in clinical trials in adult AML patients, while BsAbs targeting PR-1 and CLL-1 are in preclinical evaluation [36]. The success of blinatumomab-targeting CD19 in pediatric ALL indicates that similar success can be achieved for pediatric AML once an ideal target is identified. Our data showing the preclinical efficacy of a MSLN-targeting scFv BsAb provides preclinical validation for the use of immunotherapy against MSLN, a recently identified pediatric AML target.

## Data Availability

Data presented in this article is available in here and in Supplementary Materials.

## Supplementary Materials

The following are available online at https://www.mdpi.com/article/10.3390/cancers13235964/s1. Table S1: Amino acid sequences for all constructs in this study; Figure S1: Expression and purification of two scFv-scFv T cell engagers targeting MSLN; Figure S2.Efficacy of MSLN-targeting BsAbs in MV4;11:MSLN xenograft model; Figure S3. MSLN-targeting BsAbs in NTPL-146 model.
