# Supplementary material for: Immunotherapeutic Targeting of Mesothelin Positive Pediatric AML Using Bispecific T Cell Engaging Antibodies
Source: Cancers (Basel). 2021 Nov 26;13(23):5964. doi: 10.3390/cancers13235964 (PMC8657033; doi:10.3390/cancers13235964)
Supplement: Supplementary file 1 [file cancers-13-05964-s001.zip › cancers-1467108-supplementary.pdf]

Immunotherapeutic Targeting of Mesothelin Positive Pediatric AML Using Bispecific T Cell Engaging Antibodies

Anilkumar Gopalakrishnapillai [1,†], Colin E. Correnti [2,†], Kristina Pilat [2], Ida Lin [2], Albe Man Kid Chan [2], Ashok D. Bandaranayake [2], Christopher Mehlin [2], Anne Kisielewski [1], Darcy Hamill [1], Allison J. Kaeding [2], Soheil Meshinchi [2], James M. Olson [2], E. Anders Kolb [1] and Sonali P. Barwe [1,*]

Table S1. Amino acid sequences for all constructs in this study

BsAb	Format	Cancer cell-targeting domain	CD3-targeing domain
MSLN^{AMA}-CD3^{L2K}	scFv-scFv	AMA - Amatuximab	L2K - Blinatumomab
MSLN^{AMA}-CD3^{AMG}	scFv-scFv	AMA - Amatuximab	AMG – AMG 330
CD19-CD3^{L2K}	scFv-scFv	AMA - Blinatumomab	L2K - Blinatumomab
MSLN^{AMA}-CD3^{L2K}	IgG-scFv	AMA - Amatuximab	L2K - Blinatumomab

Amino acid sequences for all constructs in this study

>scFv-scFv MSLN^{AMA}-CD3^{L2K} (MDT000481)

```
METDTLLLWVLLLWVPGSTGDIELTQSPAIMSASPGEKVTMTCSASSSVSYMHWYQQKSGTSPK
RWIYDTSKLASGVPGRFSGSGSGNSYSLTISSVEAEDDATYYCQQWSKHPLTFGSGTKVEIKGG
GGSGGGGSGGGGSQVQLQQSGPELEKPGASVKISCKASGYSFTGYTMNWVKQSHGKSLEWIGLI
TPYNGASSYNQKFRGKATLTVDKSSSTAYMDLLSLTSEDSAVYFCARGGYDGRGFDYWGSGTPV
TVSSGGGGSDIKLQQSGAELARPGASVKMSCKTSGYTFTRYTMHWVKQRPGQGLEWIGYINPSR
GYTNYNQKFKDKATLTTDKSSSTAYMQLSSLTSEDSAVYYCARYYDDHYCLDYWGQGTTLTVSS
VEGGSGGSGGSGGSGGVDDIQLTQSPAIMSASPGEKVTMTCRASSSVSYMNWYQQKSGTSPKRW
IYDTSKVASGVPYRFSGSGSGTSYSLTISSMEAEDAATYYCQQWSSNPLTFGAGTKLELKHHHH
HH
```

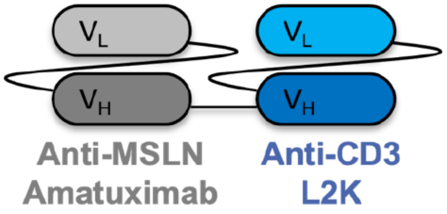

>scFv-scFv MSLN^{AMA}-CD3^{AMG} (MDT000482)
METDTLLLWVLLLWVPGSTGDIELTQSPAIMSASPGEKVTMTCSASSSVSYMHWYQQKSGTSPK
RWIYDTSKLASGVPGRFSGSGSGNSYSLTISSVEAEDDATYYCQQWSKHPLTFGSGTKVEIKGG
GGSGGGGSGGGGSQVQLQQSGPELEKPGASVKISCKASGYSFTGYTMNWVKQSHGKSLEWIGLI
TPYNGASSYNQKFRGKATLTVDKSSSTAYMDLLSLTSEDSAVYFCARGGYDGRGFDYWGSGTPV
TVSSGGGGSEVQLVESGGGLVQPGGSLKLSCAASGFTFNKYAMNWVRQAPGKGLEWVARIRSKY
NNYATYYADSVKDRFTISRDDSKNTAYLQMNNLKTEDTAVYYCVRHGNFGNSYISYWAYWGQGT
LVTVSSGGGGSGGGGSGGGGSQTVVTQEPSLTVSPGGTVTLTCGSSTGAVTSGNYPNWVQQKPG
QAPRGLIGGTKFLAPGTPARFSGSLLGGKAALTLSGVQPEDEAEYYCVLWYSNRWVFGGGTKLT
VLHHHHHH

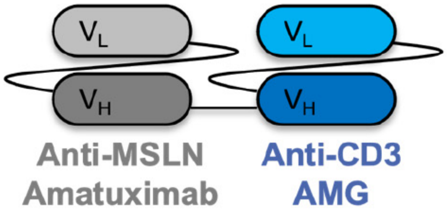

>IgG-scFv MSLN^{AMA}-CD3^{L2K} Heavy Chain(MDT000619)

MGWSCIILFLVATATGVHSQVQLQQSGPELEKPGASVKISCKASGYSFTGYTMNWVKQSHGKSL
EWIGLITPYNGASSYNQKFRGKATLTVDKSSSTAYMDLLSLTSEDSAVYFCARGGYDGRGFDYW
GSGTPVTVSSASTKGPSVFPLAPSSKSTSGGTAALGCLVKDYFPEPVTVSWNSGALTSGVHTFP
AVLQSSGLYSLSSVVTVPSSSLGTQTYICNVNHKPSNTKVDKKVEPKSCDKTHTCPPCPAPELL
GGPSVFLFPPKPKDTLMISRTPEVTCVVVDVSHEDPEVKFNWYVDGVEVHNAKTKPREEQYAST
YRVVSVLTVLHQDWLNGKEYKCKVSNKALPAPIEKTISKAKGQPREPQVYTLPPSRDELTKNQV
SLTCLVKGFYPSDIAVEWESNGQPENNYKTTPPVLDSDGSFFLYSKLTVDKSRWQQGNVFSCSV
MHEALHNHYTQKSLSLSPGK

>IgG-scFv MSLN^{AMA}-CD3^{L2K} Light Chain(MDT000620)
METDTLLLWVLLLWVPGSTGDIELTQSPAIMSASPGEKVTMTCSASSSVSYMHWYQQKSGTSPK
RWIYDTSKLASGVPGRFSGSGSGNSYSLTISSVEAEDDATYYCQQWSKHPLTFGSGTKVEIKRT
VAAPSVFIFPPSDEQLKSGTASVVCLLNNFYPREAKVQWKVDNALQSGNSQESVTEQDSKDSTY
SLSSTLTLSKADYEKHKVYACEVTHQGLSSPVTKSFNRGECGGGGSGGGGSGGGGSDIKLQQSG
AELARPGASVKMSCKTSGYTFTRYTMHWVKQRPGQGLEWIGYINPSRGYTNYNQKFKDKATLTT
DKSSSTAYMQLSSLTSEDSAVYYCARYYDDHYCLDYWGQGTTLTVSSVEGGSGGSGGSGGSGGV
DDIQLTQSPAIMSASPGEKVTMTCRASSSVSYMNWYQQKSGTSPKRWIYDTSKVASGVPYRFSG
SGSGTSYSLTISSMEAEDAATYYCQQWSSNPLTFGAGTKLELKHHHHHH

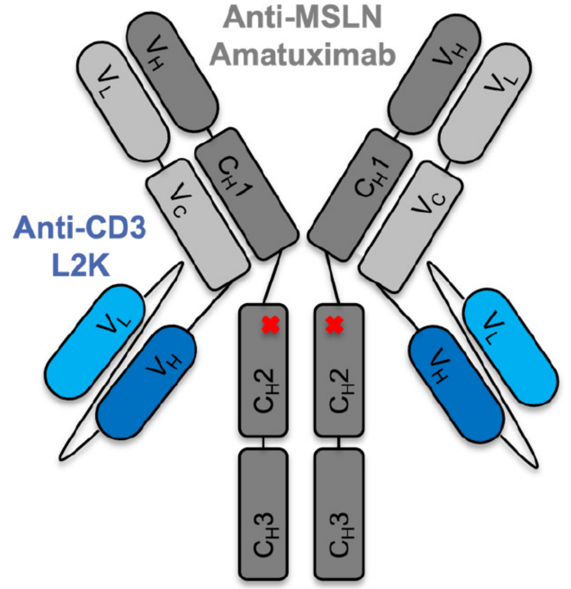

>scFv-scFv CD19-CD3 (MDT000244)

<u>METDTLLLWVLLLWVPGSTG</u>DIQLTQSPASLAVSLGQRATISCKASQSVDYDGDSYLNWYQQIP
GQPPKLLIYDASNLVSGIPPRFSGSGSGTDFTLNIHPVEKVDAATYHCQQSTEDPWTFGGGTKL
EIKGGGGSGGGGSGGGGSQVQLQQSGAELVRPGSSVKISCKASGYAFSSYWMNWVKQRPGQGLE
WIGQIWPGDGDTNYNGKFKGKATLTADESSSTAYMQLSSLASEDSAVYFCARRETTTVGRYYYA
MDYWGQGTTVTVSSGGGGSDIKLQQSGAELARPGASVKMSCKTSGYTFTRYTMHWVKQRPGQGL
EWIGYINPSRGYTNYNQKFKDKATLTTDKSSSTAYMQLSSLTSEDSAVYYCARYYDDHYCLDYW
GQGTTLTVSSVEGGSGGSGGSGGSGGVDDIQLTQSPAIMSASPGEKVTMTCRASSSVSYMNWYQ
QKSGTSPKRWIYDTSKVASGVPYRFSGSGSGTSYSLTISSMEAEDAATYYCQQWSSNPLTFGAG
TKLELKHHHHHH

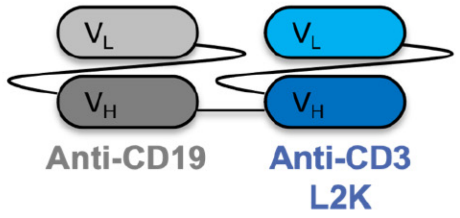

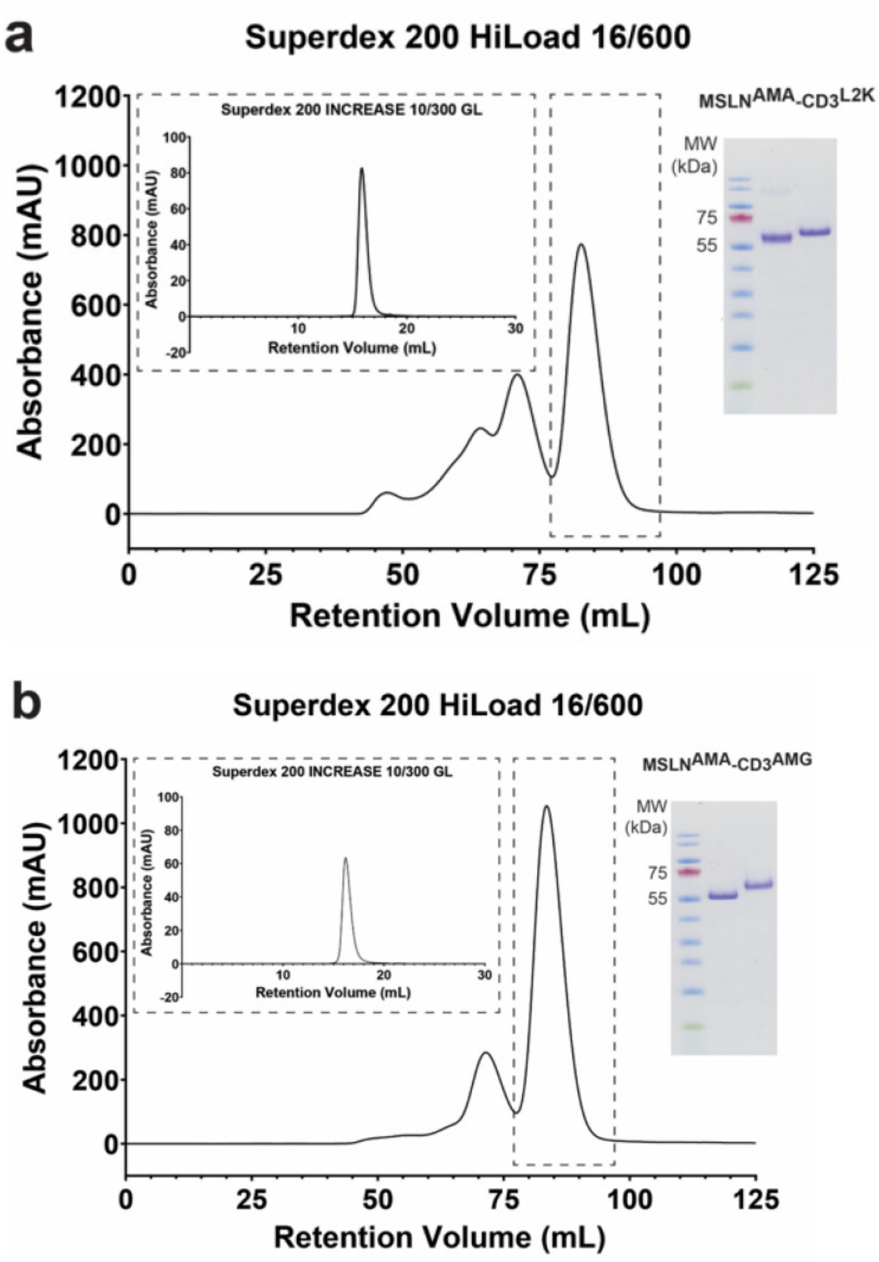

Figure S1. Expression and purification of two scFv-scFv T cell engagers targeting MSLN. **a**, SEC and non-reducing, reducing SDS-PAGE analyses of the scFv-scFv BsAb derived from the sequences of amatuximab and blinatumomab. The main SEC chromatogram shows the elution profile of the protein following NiNTA affinity purification and the dashed rectangle highlights pooled fractions. Left SEC inset shows the final purified protein following a single freeze, thaw cycle. Right SDS-PAGE gel inset shows the final purified protein under non-reducing and reducing conditions. The change in mobility under reducing conditions is due to the dissolution of intrachain disulfides in the scFv-scFv construct. **b**, SEC and nonreducing, reducing SDS-PAGE analyses of the scFv-scFv BsAb derived from the sequences of amatuximab and AMG 330 (SEQID 100 USPTO) as described in a.

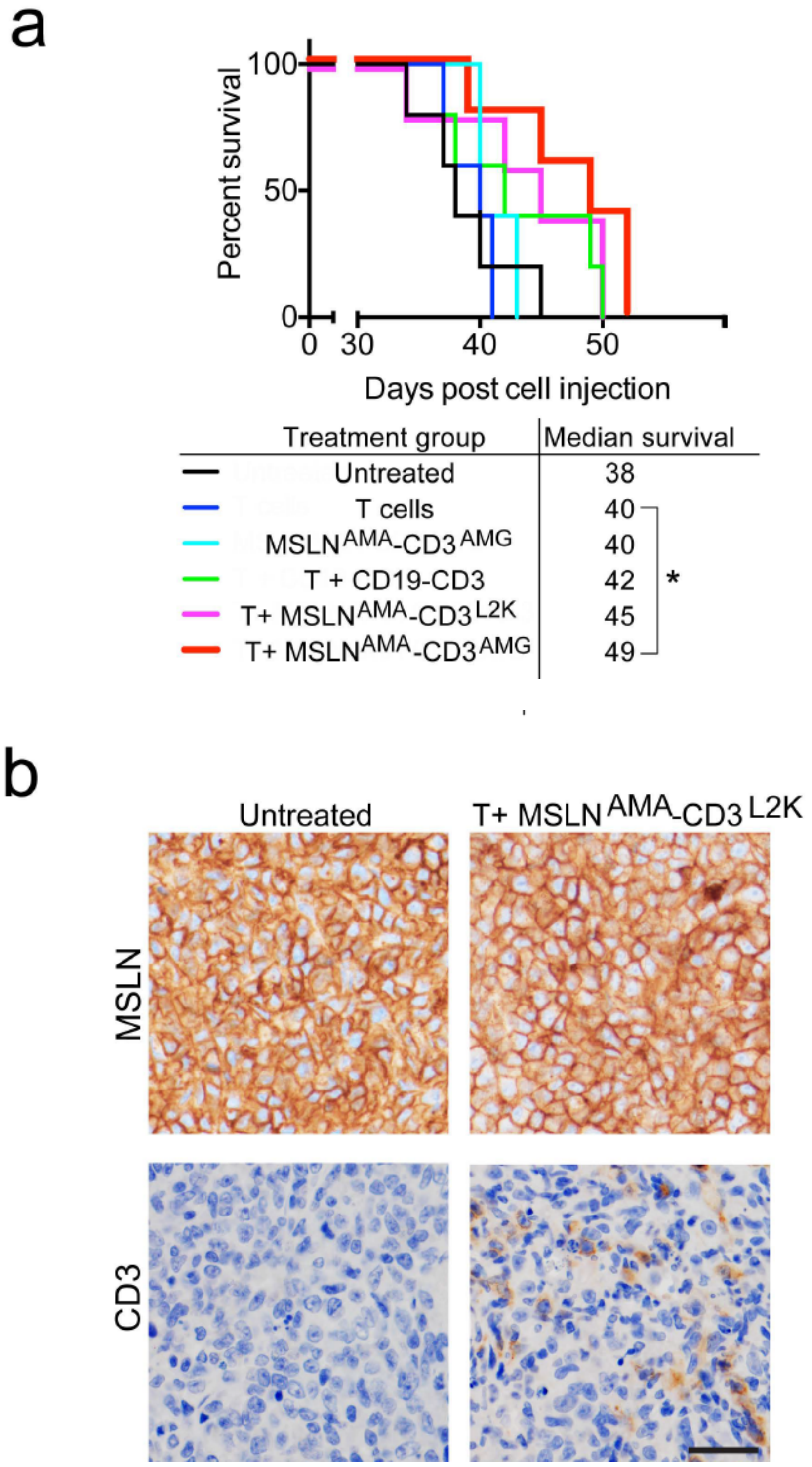

Figure S2. Efficacy of MSLN-targeting BsAbs in MV4;11:MSLN xenograft model. **a**, Kaplan-Meier survival plot showing the median survival of MV4;11:MSLN engrafted mice receiving indicated treatments. *P<0.05. **b**, Immunohistochemical staining of extramedullary chloromas with anti-MSLN (Abcam) or anti-CD3 (BioLegend) antibodies. Scale bar=20 microns.

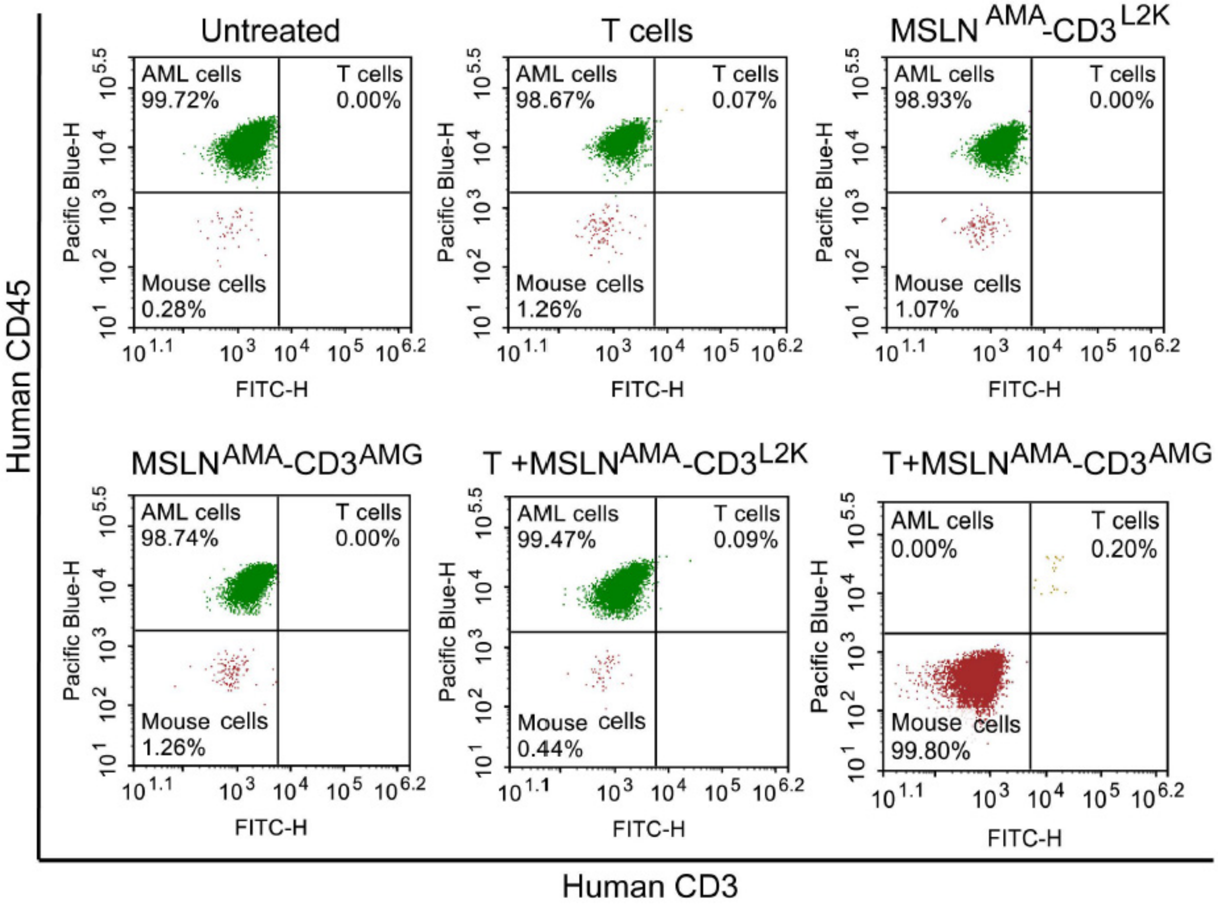

Figure S3. MSLN-targeting BsAbs in NTPL-146 model. Mice were euthanized when they reached pre-determined experimental endpoints. Bone marrow was. flushed and stained with mouse specific CD45-APC, human specific CD45-Pacific blue and human specific CD3-FITC (OKT3 or L2K) antibodies. Representative flow plots showing the terminal bone marrow AML load and T cell counts are presented. The CD45+CD3- cells were labeled as AML cells and the CD45+CD3+ cells were labeled as T cells.
